# Supplementary material for: Comparative brain-wide mapping of ketamine- and isoflurane-activated nuclei and functional networks in the mouse brain
Source: eLife. 2024 Mar 21;12:RP88420. doi: 10.7554/eLife.88420 (PMC10957177; doi:10.7554/eLife.88420)
Supplement: Figure 3—source data 1. [file elife-88420-fig3-data1.docx]

**Figure 3—source data 1. Summary of prior studies on KET activated brain regions identified via c-Fos immunostaining.**

| **Brain regions** | **Function** | **Treatment Regimen** | **Duration** | **Time of day** | **Effect** | **References** |
| --- | --- | --- | --- | --- | --- | --- |
| CeA | Pain Suppression | 100mg/kg and 10mg/kgxylazine i.p. | 2h | / | Up-regulated | Hua T, et al.,(2020) |
| SON | Natural sleep/General anesthesia | 100mg/kg with or without xylazine(10mg/kg) i.p | 2h | / | Up-regulated | Jiang-Xie LF, et al.,(2019) |
| pCC | / | 100mg/kg i.p. | 2h | / | Up-regulated | Nagata A, et al.,(1998) |
| pCC, RSC | / | 100mg/kg i.p. | 2h | / | Up-regulated | Nagata A, et al.,(2001) |
| RSP, CeA, Midline thalamic nuclei | Antidepressant | 10mg/kg i.p. | 1.5h | / | Up-regulated | Inta D, et al.,(2009) |
| Cg, BF, Orx, TMN, vlPAG, LC, LHb, EW, midline thalamus | LC/vPAG engaged in analgesia | 150mg/kg, 60mg/kg, 30mg/kg i.p. | 2h | ZT3 | Up-regulated | Lu J, et al.,(2008) |
| CC, PVT | / | 100mg/kg i.p. | / | / | / | Nakao S, et al.,(1993) |
| PC, RSP | / | 50mg/kg i.p. | 2h | ZT3-9 | Up-regulated | Nakao S, et al.,(2002) |

Abbreviation: CeA, central amygdalar nucleus; SON, supraoptic nucleus; pCC, posterior cingulate cortex; RSC/RSP, retrosplenial area; Cg, cingulate cortex; BF, basal forebrain cholinergic cells; Orx, perifornical orexin cells; vlPAG, ventrolateral periaquductal gray dopamine cells; LC, locus coeruleus; LHb, lateral habenular nucleus; EW, Edinger-Westphal nucleus; CC, cerebral cortex; PVT, paraventricular nucleus of the thalamus; PC, posterior cingulate. RSP, retrosplenial area.
