# Supplementary material for: Comparative brain-wide mapping of ketamine- and isoflurane-activated nuclei and functional networks in the mouse brain
Source: eLife. 2024 Mar 21;12:RP88420. doi: 10.7554/eLife.88420 (PMC10957177; doi:10.7554/eLife.88420)
Supplement: Figure 4—source data 1. [file elife-88420-fig4-data1.docx]

**Figure 4—source data 1. Summary of prior studies on brain regions activated by ISO, as detected through c-Fos immunostaining.**

| **Brain regions** | **Function** | **Concentration of ISO** | **Duration** | **Time of day** |  | **References** |
| --- | --- | --- | --- | --- | --- | --- |
| VLPO | Sleep-Promoting, Hypnosis | 1.2%, 0.6%, 0.3% | 2h | ZT12-14/ZT4-6 | Up-regulated | Moore JT,.et al.,(2012) |
| PIR, LSD, LSV | / | 4% 5mins+1% | 2h | ZT2-7 | Up-regulated | Smith ML, et al.,(2016) |
| NTS, VeN | Vomiting | 1% and 3% | 1.5h | / | Up-regulated | Gupta RG, et al.,(2016) |
| CeA | Pain Suppression | 1.5% | 2h | / | Up-regulated | Hua T, et al.,(2022) |
| SON | Natural sleep, General anesthesia | 1%,1.2% | 2h | / | Up-regulated | Jiang-Xie LF, et al.,(2019) |
| VLPO, MnPO | Sleep and unconsciousness | 1.2% | 2h | ZT14-17 | Up-regulated | Han B, et al.,(2014) |
| EW, VLPO, LC | Sleep-Promoting, analgesia | 2% | 2h | ZT12 | Up-regulated | Lu J, et al.,(2008) |
| AP | Nausea, vomiting | 1.3%,2.6% | 2h | / | Up-regulated | Hase T, et al.,(2019) |

Abbreviation: VLPO, ventrolateral preoptic nucleus; PIR, piriform area; LSD, lateral septal nucleus, dorsal part; LSV, lateral septal nucleus, ventral part; NTS, nucleus of the solitary tract; VeN, ventral group of the dorsal thalamus; CeA, central amygdalar nucleus; SON, supraoptic nucleus; MnPO, median preoptic nucleus; EW, Edinger-Westphal nucleus; LC, locus coeruleus; AP, area postrema.
